# Dissemination of the Flavivirus Subgenomic Replicon Genome and Viral Proteins by Extracellular Vesicles

**DOI:** 10.3390/v16040524

**Published:** 2024-03-28

**Authors:** Tomohiro Ishikawa, Kentaro Narita, Kinichi Matsuyama, Michiaki Masuda

**Affiliations:** 1 Department of Microbiology, Dokkyo Medical University School of Medicine, 880 Kita-kobayashi, Mibu 321-0293, Tochigi, Japanm-masuda@dokkyomed.ac.jp (M.M.); 2 Department of Pathology, Dokkyo Medical University Hospital, 880 Kita-kobayashi, Mibu 321-0293, Tochigi, Japan

**Keywords:** Japanese encephalitis virus, dengue virus, extracellular vesicle, exosome, replicon

## Abstract

Extracellular vesicles (EVs) such as exosomes have been shown to play physiological roles in cell-to-cell communication by delivering various proteins and nucleic acids. In addition, several studies revealed that the EVs derived from the cells that are infected with certain viruses could transfer the full-length viral genomes, resulting in EVs-mediated virus propagation. However, the possibility cannot be excluded that the prepared EVs were contaminated with infectious viral particles. In this study, the cells that harbor subgenomic replicon derived from the Japanese encephalitis virus and dengue virus without producing any replication-competent viruses were employed as the EV donor. It was demonstrated that the EVs in the culture supernatants of those cells were able to transfer the replicon genome to other cells of various types. It was also shown that the EVs were incorporated by the recipient cells primarily through macropinocytosis after interaction with CD33 and Tim-1/Tim-4 on HeLa and K562 cells, respectively. Since the methods used in this study are free from contamination with infectious viral particles, it is unequivocally indicated that the flavivirus genome can be transferred by EVs from cell to cell, suggesting that this pathway, in addition to the classical receptor-mediated infection, may play some roles in the viral propagation and pathogenesis.

## 1. Introduction

Genus *Flavivirus* consists of more than 70 members including important pathogens such as Japanese encephalitis virus (JEV) and dengue virus (DENV) [1]. Flaviviruses are distributed worldwide and billions of people are at risk for the diseases caused by flavivirus infection [2]. Flavivirus infection leads to various symptoms ranging from mild flu-like illness to severe diseases, such as meningitis and encephalitis for JEV and hemorrhagic fever for DENV [3]. However, the pathological mechanisms of the diseases caused by flaviviruses have not been fully elucidated.

JEV and DENV are arthropod-borne enveloped viruses, which approximately contain 11-kb positive-strand RNA genomes. The viral genome encodes three structural proteins, namely capsid, precursor membrane (prM), and envelope (E), as well as seven nonstructural (NS) proteins, namely NS1, NS2A, NS2B, NS3, NS4A, NS4B, and NS5 [4]. The infectious virion is approximately 50–70 nm in diameter and contains mature M and E proteins on its surface. The E protein contains the receptor-binding domain and the fusion peptide. The NS proteins are known to be involved in viral replication and also modulation of host immune responses [5]. For example, the NS5 protein possesses a guanylyl transferase/methyltransferase domain and an RNA-dependent RNA polymerase domain essential for viral genome replication, while it is known to block type I interferon signaling as well [5].

Extracellular vesicles (EVs) have been known to contain structures such as exosomes and microvesicles. Exosomes are extracellular vesicles sized approximately 40–100 nm in diameter and are formed within a multivesicular endosome (MVE) and released when the MVE is fused with the plasma membrane, whereas microvesicles are extracellular vesicles sized 50–1000 nm in diameter and formed by budding from the plasma membrane [6]. Most of the cell types are known to produce and release EVs. When EVs were originally discovered in the 1980s, their functions were unknown. However, recent studies have demonstrated that EVs bear a variety of physiological and pathological functions. For instance, EVs carry membrane proteins, intra-cellular proteins, extra-cellular proteins, DNA, and RNA and play roles in cell-to-cell communications by delivering those cargoes [7]. It has also been reported that EVs are involved in tumor growth and tumor metastasis [8,9]. In addition, recent studies have revealed that EVs are involved in the infection of certain viruses by transferring viral components and host cellular factors [10]. For example, exosomes produced by the cells infected with human immunodeficiency virus (HIV), human T-cell leukemia virus 1, or hepatitis C virus (HCV) were shown to deliver viral proteins [11,12,13]. It has also been reported that EVs can deliver viral genomes of HIV, HCV, human pegi virus, hepatitis A virus, and enterovirus, contributing to virus dissemination [13,14,15,16,17]. Furthermore, it has been shown that EVs, which contain cellular factors, such as miRNA and proteins, could affect immune responses, suggesting that EVs might be involved in viral pathogenesis [18]. These findings imply that virus propagation could be mediated by EVs in addition to the classical receptor-mediated infection. As to flavivirus, several studies have suggested that its genome RNA could be transferred by EVs from cell to cell [19,20,21]. However, those studies utilized the EVs derived from virus-infected cells, raising the possibility that the EV samples may have been contaminated with infectious viral particles.

To exclude this possibility, the cells that harbor subgenomic replicon derived from JEV and DENV without producing any replication-competent virus were employed in this study as the EV donor. Since the replicon carries the luciferase gene, the transfer of the replicon RNA could be quantitatively evaluated by measuring the luciferase activity in the recipient cells. The results indicated that the EVs produced by the replicon-harboring cells contained the replicon RNA, which could be transferred to other cells of various types and replicated in the recipient cells. In addition, the EVs were found to contain viral proteins including the non-structural proteins that are not normally found in the viral particles. Among those, DENV NS1 proteins were shown to exist inside the EVs. This is the first study that unequivocally demonstrated EV-mediated cell-to-cell transfer of flaviviral RNA. The results suggest that EV-mediated transfer of the viral components, in addition to the classical receptor-mediated infection, may play previously unknown roles in the propagation and pathogenesis of flaviviruses.

## 2. Materials and Methods

### 2.1. Cells and Viruses

Vero (monkey kidney), HeLa (human cervix adenocarcinoma), PK15 (porcine kidney), and SK-N-SH (human neuroblastoma) cells were grown at 37 °C with 5% CO_2_ in Eagle’s minimum essential media (MEM), supplemented with 10% fetal bovine serum (FBS), 10 mM nonessential amino acid (NEAA; Thermo Fisher Scientific, Waltham, MA, USA), and penicillin (1000 unit/mL)-streptomycin (100 µg/mL) (PS). BHK (hamster kidney) and HepG2 (human hepatoma) cells were grown at 37 °C with 5% CO_2_ in Dulbecco’s modified MEM supplemented with 10% FBS, NEAA, and PS. K562 (human leukemia) cells were grown at 37 °C with 5% CO_2_ in RPMI1640 media supplemented with 10% FBS. C6/36 (*Aedes albopictus* mosquito) cells were grown at 30 °C with 5% CO_2_ in MEM supplemented with 10% FBS, NEAA, and PS. JEV of the Muar strain and DENV of the New Guinea C (NGC) strain were propagated in C6/36 as described previously [22]. Following virus infection and EV inoculation, cells were maintained in MEM supplemented with 1% FBS, NEAA, and 10 mM HEPES (MEM-1%FBS).

### 2.2. Establishment of Subgenomic Replicon Harboring Cell Lines

**Figure 1:**
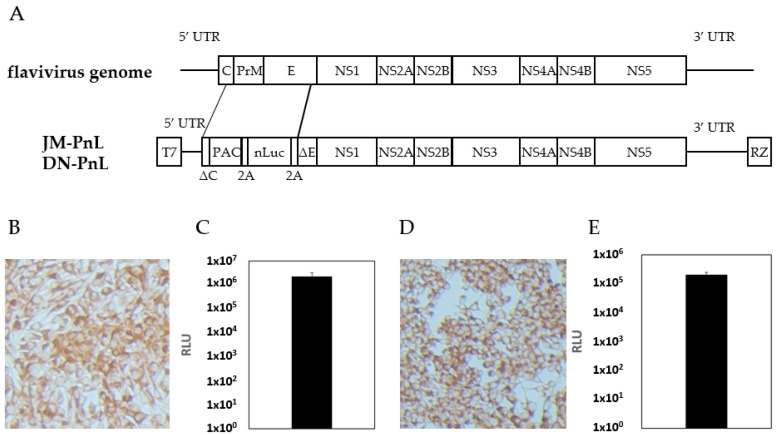
Establishment of JEV- and DENV-subgenomic replicon harboring cells. (**A**) Structures of the flavivirus genome, JM-PnL, and DN-PnL were shown. UTR: untranslated region, RZ: hepatitis delta virus derived ribozyme. (**B**) Immunostaining of BHK/JM-PnL cells. Cells were stained with anti-NS1 antibody. (**C**) Luciferase activities of BHK/JM-PnL cells (*n* = 2). (**D**) Immunostaining of BHK/DN-PnL cells. Cells were stained with anti-NS1 antibody (*n* = 2). (**E**) Luciferase activities of BHK/DN-PnL cells.

To construct the subgenomic replicon, JM-PnL (Figure 1), the *puromycin N-acetyl-transferase* (*PAC*), and the deep sea shrimp-derived *nano-luciferase* (*nLuc*) genes were individually amplified by PCR and the obtained DNA fragments were inserted into the previously reported JEV Muar strain-based replicon clone [23]. A similar strategy was applied to construct the DENV NGC-derived replicon, DN-PnL (Figure 1), bearing the PAC and nLuc genes. The obtained clones of plasmid DNA were linearized by *Swa* I digestion and then subjected to in vitro RNA transcription using a MEGAscript T7 Kit (Thermo Fisher Scientific). The prepared replicon RNAs were electroporated into BHK cells by using the GENE PULSER II (Bio Rad, Hercules, CA, USA) at 500 V and 25 µF [22]. To select the cells successfully harboring the replicon RNA, puromycin was added at 10 µg/mL from the next day and the surviving cells were designated as BHK/JM-PnL and BHK/DN-PnL, respectively.

### 2.3. Immunostaining

Expression of the viral proteins was detected by immunostaining as described previously [22]. Briefly, cells fixed with 50% acetone–50% methanol solution were serially incubated with the 6H4 anti-NS1 mouse monoclonal antibody [24], biotinylated anti-mouse IgG (Vector Laboratories, Burlingame, CA, USA), and peroxidase-conjugated avidin-biotin complex (Vector Laboratories). The cells with virus protein expression were visualized by incubation with the peroxidase substrate, 0.02% 3,3′-diaminobenzidine in 0.008% H_2_O_2_.

### 2.4. EV Sample Preparation

The BHK/JM-PnL and BHK/DN-PnL cells in the culture plates were rinsed with PBS and then cultured in 10% VP-SFM (Thermo Fisher Scientific)–90% MEM supplemented with NEAA (VPSFM-MEM). Then, the culture supernatants were collected daily and subjected to centrifugation at 3000 rpm for 5 min to remove cell debris. The cleared culture fluids were concentrated by about 100-fold with the Amicon Ultra 100 K (Merck, Burlington, MA, USA). Approximately 1 mL of the concentrated culture fluids were subjected to EV isolation by using the MagCapture™ exosome isolation kit PS (FUJIFILM Wako Pure Chemical Corporation, Osaka, Japan) according to the manufacturer’s instruction except that the addition of the beads was repeated three times for each culture fluid preparation. All of the EVs eluted from the three batches of beads were combined and used as the EV sample derived from each fluid preparation. After EV isolation with the beads, the remaining concentrated culture fluids were used as EV-depleted samples in some analyses.

In some experiments where EV production needs to be inhibited, GW4869 (7.5 µM in DMSO; Cayman Chemical, Ann Arbor, MI, USA) was added 24 h before changing the media to VPSFM-MEM, which also contained GW4869.

### 2.5. Electron Microscopy

Five microliters of the isolated EV sample were applied on a formvar-coated grid (Nisshin EM Co., Ltd., Tokyo, Japan) and incubated for 2 min at room temperature. The grid was stained with 5% uranyl acetate for 30 s at room temperature. The images were obtained by the transmission electron microscope HT7800 (Hitachi High-Tech Corporation, Tokyo, Japan). The diameter of the observed EVs was measured (*n* = 20). 

### 2.6. EV Inoculation and Luciferase Assay

The prepared EVs and the EV-depleted culture supernatants were diluted in VPSFM-MEM 20-fold and then inoculated onto the target cells that had been seeded in a 96-well plate and rinsed with PBS before the inoculation. In some experiments, the EVs incubated in 0.5 mg/mL of Triton X-100 (Tx-100) at room temperature for 5 min were diluted by 30-fold in VPSFM-MEM and then inoculated onto BHK cells. In some other experiments, the EVs were treated before inoculation with 5 µg/mL RNase A (Nippon Gene, Tokyo, Japan) for 15 min at 37 °C and diluted in VPSFM-MEM by 20-fold.

After inoculation with the EVs, the cells were incubated at 37 °C for 4 h. Then, the inoculum was removed and the cells were maintained in MEM-1%FBS for 72 h. In some experiments, the cells were maintained in MEM-1%FBS in the presence of 300 µM ribavirin (FUJIFILM Wako Pure Chemical Corporation). At 72 h, the cells were rinsed with PBS and lysed with the cell lysis buffer supplied in the Nano-Glo Luciferase Assay System (Promega, Madison, WI, USA) and the luciferase activities were measured by the same Assay System according to the manufacturer’s instructions [23]. The luminescence was measured by a Lumat LB9501 (Berthold Technologies, Bad Wilbad, Germany).

### 2.7. PCR

The total RNA contained in the isolated EVs was purified by an EV RNA purification system (System Biosciences, Palo Alto, CA, USA), according to the manufacturer’s instructions. Approximately 40 ng of RNA was reverse transcribed by ReverTra Ace (TOYOBO, Tokyo, Japan) with the NS5 gene-specific primers, namely, 5′-agctcttgtacgcgttccgat-3′ and 5′-ctccttctccctccatctgtc-3′ for JEV Muar and DENV NGC strains, respectively, according to the manufacturer’s instructions. By using 3 µL of the obtained cDNA solution, PCR amplification was carried out by KOD Ver 2 (TOYOBO) according to the manufacturer’s instructions. For amplifying the nLuc gene, oligonucleotide 5′-ggtgatcctgcactatgg-3′ was used as the sense primer. As the antisense primers, oligonucleotides 5′-tcaaccacactcgagtcgacg-3′ and 5′-gagcggattccacaaatgccc-3′, which correspond to the adjacent viral sequences of JM-PnL and DN-PnL, respectively, were used. For amplifying the viral NS5 gene, oligonucleotides 5′-tggcagcaagtccct-3′ and 5′- agctcttgtacgcgttccgat-3′ were used as sense and antisense primers, respectively, for the JEV Muar strain. Similarly, oligonucleotides 5′-ggtgagaagcaatgcagcctt-3′ and 5′-tccataggtaccaacttgtcc were used for amplifying the NS5 gene of the DENV NGC strain.

### 2.8. Real-Time PCR

Total RNAs were obtained by CellAmp™ Direct RNA Prep Kit for RT-PCR (TaKaRa Bio Inc., Shiga, Japan) from C6/36 cells in a 96-well plate inoculated with EVs derived from BHK/JM-PnL and BHK/DN-PnL cells. Total RNAs of the EVs treated with RNase A in the presence/absence of Tx-100 were isolated by a microRNA Extractor^®^ Kit for Purified EV (FUJIFILM Wako Pure Chemical Corporation). JM-PnL and DN-PnL replicon genomes were quantified by RNA-direct™ SYBR^®^ Green Realtime PCR Master Mix (TOYOBO). Primers used for JM-PnL were sense, 5′-ccctcagaaccgtctcggaa-3′, and anti-sense, 5′-ctattcccaggtgtcaatatgctgt-3′, and primers used for DN-PnL were sense, 5′-agttgttagtctacgtggaccga-3′, and anti-sense, 5′-cgcgtttcagcatattgaaag-3′. The real-time PCR was carried out by Thermal Cycler Dice Real-Time System III (Takara Bio Inc.). The in vitro-transcribed JM-PnL and DN-PnL were used as standard.

### 2.9. Treatment of the Cells with Antibodies and Inhibitors

For treatment with antibodies, HeLa or K562 cells were incubated with anti-CD33 (15 µg/mL; Southern Biotech, Birmingham, AL, USA), anti-Tim-1 (20 µg/mL; BioLegend, San Diego, CA, USA), or anti-Tim-4 antibody (20 µg/mL; Novus Biologicals, Centennial, CO, USA) for 1 h at 37 °C. The prepared EV samples were then inoculated onto the cells in the presence of the antibodies. After incubation for 4 h at 37 °C, the inoculum was removed and the cells were maintained in MEM-1%FBS for 72 h before the luciferase assay.

For treatment with various entry inhibitors, HeLa cells prepared in the 96-well plate were incubated with Pitstop 2 (30 µM; Merck), LY294002 (5 µM; FUJIFILM Wako Pure Chemical Corporation), dynasore (80 µM; Tokyo Chemical Industry Co., Tokyo, Japan), genistein (150 µM; FUJIFILM Wako Pure Chemical Corporation), or 5-[N-ethyl-N-isopropyl] amiloride (EIPA: 100 µM; Cayman Chemical) for 30 min at 37 °C. The prepared EV samples were then inoculated onto the cells in the presence of entry inhibitors. Four hours later, the cells were rinsed with 0.5 M NaCl–0.2 M acetic acid solution and then maintained in MEM–1%FBS for 72 h before the luciferase assay.

### 2.10. Western Blot

The EVs, culture supernatants derived from BHK/JM-PnL and BHK/DN-PnL cells and EV-depleted culture supernatants added with the NuPAGE LDS sample buffer (Thermo Fisher Scientific) containing 100 mM dithiothretiol were fractionated on 4–12% gradient polyacrylamide gels (NuPAGE; Thermo Fisher Scientific) in the NuPAGE MOPS SDS Running Buffer (Thermo Fisher Scientific) for 80 min at 150 V and then were transferred to Immobilon-P membranes (Merck) in the NuPAGE Transfer Buffer (Thermo Fisher Scientific) for 80 min at 30 V. The membranes were treated with the blocking buffer containing 0.05% Tween 20 and 5% skim milk in 50 mM Tris-buffered saline (pH 8.0) and serially incubated with anti-JEV (NS1, NS2B, NS3, NS4B, and NS5) or anti-DENV (NS1, NS2B, NS3, NS4B, and NS5) rabbit monoclonal antibodies (Gene Tex, Irvine, CA, USA), horseradish peroxidase-conjugated anti-rabbit IgG (Cytiva, Tokyo, Japan), and Lumi-Light Plus (Merck). To detect luminescence, the membranes were examined by LuminoGraph I (ATTO, Tokyo, Japan).

### 2.11. Proteinase K Protection Assay

EVs were incubated with proteinase K (100 µg/mL) in the presence/absence of Tx-100 (0.5%) for 30 min on ice and then phenylmethylsulfonyl fluoride (2 mM) was added. Those samples were subjected to Western blot as described above.

### 2.12. Statistical Analysis

Statistical analysis of the data was performed with the Student’s *t*-test. For multiple comparisons, the Holm–Bonferroni method was used for correction. Differences were considered statistically significant when the *p*-value was less than 0.05.

## 3. Results

### 3.1. Establishment of JEV and DENV Subgenomic Replicon Expressing Cells

To establish subgenomic replicon harboring cells as EV donor cells, DNA clones of the *nLuc* gene-bearing replicons JM-PnL and DN-PnL were constructed from the infectious clones of JEV (Muar strain) and DENV (NGC strain), respectively (Figure 1A). The DNA clones were used as templates of in vitro transcription of the replicon RNA, which was then electroporated into BHK cells. Successful introduction of the replicon was confirmed by the detection of the replicon-coded JEV and DENV proteins and of luciferase activity (Figure 1B–E). The obtained BHK cells harboring JM-PnL and DN-PnL were designated as BHK/JM-PnL and BHK/DN-PnL, respectively.

### 3.2. Incorporation of the Replicon Genome and Viral Proteins in the EVs

**Figure 2:**
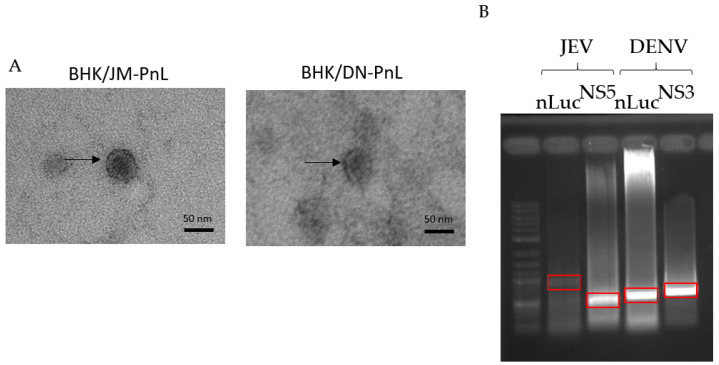
Detection of subgenomic replicon RNA within EVs. (**A**) Morphological analysis of EVs derived from BHK/JM-PnL and BHK/DN-PnL cells. Arrow indicates the typical image of EVs. Bar represents 50 nm. (**B**) Total RNAs were isolated from EVs derived from BHK/JM-PnL and BHK/DN-PnL cells. Luciferase-JEV NS1 (889 bp), JEV NS5 (464 bp), Luciferase-DENV NS1 (557 bp), and DENV NS5 (718 bp) fragments were amplified. The bands surrounded by red boxes corresponded to the expected size.

In order to elucidate the functions of EVs derived from BHK/JM-PnL and BHK/DN-PnL cells, EVs were isolated from the concentrated culture supernatants of those cells by using phosphatidyl serine affinity beads. Electron microscopy revealed that the extracellular vesicles in the prepared samples were consistent with EVs in shape (characterized as cup-shape) and size (Averages in diameter of the EVs derived from BHK/JM-PnL and BHK/DN-PnL cells were 39.6 ± 5.01 nm and 40.2 ± 5.45 nm, respectively) (Figure 2A) [25]. To examine whether the replicon genome maintained in BHK cells could be packaged in those EVs, the total RNA extracted from the EV samples was reverse transcribed and by using the obtained cDNA as a template, PCR amplification of *nLuc* and viral NS5 genes was performed. As shown in Figure 2B, the DNA fragments with expected sizes for *nLuc* and NS5 genes were amplified for both EV samples derived from BHK/JM-PnL and BHK/DN-PnL. These results suggested that the replicon genome maintained in BHK cells was associated with the EVs.

**Figure 3:**
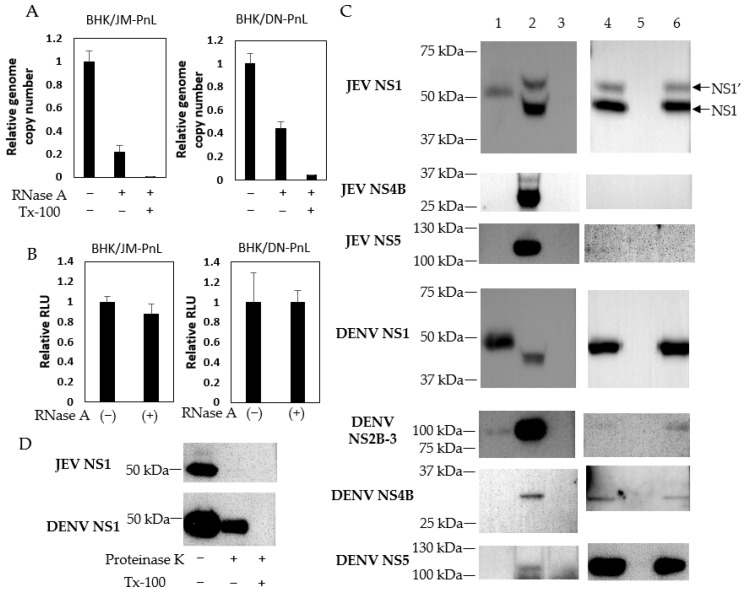
Existence of replicon RNA and viral proteins in EVs. (**A**) JM-PnL and DN-PnL replicon genomes contained in EVs treated with RNase A and/or Tx-100 were quantified by real-time PCR (*n* = 2). (**B**) BHK/JM-PnL cell- and BHK/DN-PnL cell-derived EVs treated with RNase A were inoculated onto BHK cells. Data were expressed as relative luminescence units to the negative control (*n* = 3). (**C**) Western blot analysis of isolated EVs derived from BHK/JM-PnL cells and BHK/DN-PnL cells (lane 1). JEV Muar strain and DENV2 NGC strain-infected BHK cells were used as a positive control (lane 2) and naïve BHK cells were used as a negative control (lane 3). The presence of the JEV NS1, DENV NS1, and DENV NS2B-3 in the culture supernatants derived from BHK/JM-PnL or BHK/DN-PnL cells (lane 4), BHK cells (lane 5), and EV-depleted culture supernatants (lane 6) were examined. The brightness of each picture was adjusted upon manuscript preparation. (**D**) Western blot analysis of isolated EVs treated with proteinase K in the presence/absence of Tx-100.

To analyze whether the replicon genome was packaged inside of the EVs or attached to the surface of the EVs, the EV samples were treated with RNase A in the presence/absence of Tx-100. RNase A resistant JM-PnL and DN-PnL replicon genomes were detected, whereas those were diminished when treated with RNase A in the presence of Tx-100 (Figure 3A). In addition, the RNase A treatment did not affect luciferase activities in the EVs-inoculated cells (Figure 3B). These results suggested that the replicon genome could be packaged inside of the EVs.

Whereas Western blot analysis detected viral NS1 and NS1′ proteins of approximately 45 kDa and 55 kDa, respectively, in the JEV-infected BHK cells, EVs produced by BHK/JM-PnL cells were shown to contain the protein of approximately 50 kDa reactive with the anti-NS1 antibody (Figure 3C). Similarly, EVs produced by BHK/DN-PnL cells contained the protein of approximately 50 kDa reactive with the anti-NS1 antibody while viral NS1 protein of approximately 45 kDa was detected in the DENV-infected BHK cells. In addition, the NS2B-NS3 complex of 100 kDa appeared to be incorporated in the EV produced by BHK/DN-PnL cells (Figure 3C) but not BHK/JM-PnL cells. The incorporation of other viral proteins encoded by the replicon, such as NS4B and NS5, into the EV derived from both BHK/JM-PnL and BHK/DN-PnL cells was not detected (Figure 3C).

To see if the detected viral proteins were packaged inside of the EVs or attached to the surface of the EVs, the resistance of those proteins to proteinase K in the presence/absence of Tx-100 was examined. DENV NS1 was reduced but detected after treatment with proteinase K alone, whereas DENV NS1 was diminished by treatment with proteinase K in the presence of Tx-100 (Figure 3D). Those results indicated that DENV NS1 exists both inside of the EVs and on the surface. In contrast, JEV NS1 existing inside of the EVs was not detected, thus most of the JEV NS1 seems to be attached to the surface of the EVs. Those viral proteins remained in the culture fluids after EV isolations, indicating that viral proteins associated with EVs are not the majority among viral proteins detected in the culture supernatants. 

### 3.3. Cell-to-Cell Transfer of the Replicon Genome Mediated by EVs

**Figure 4:**
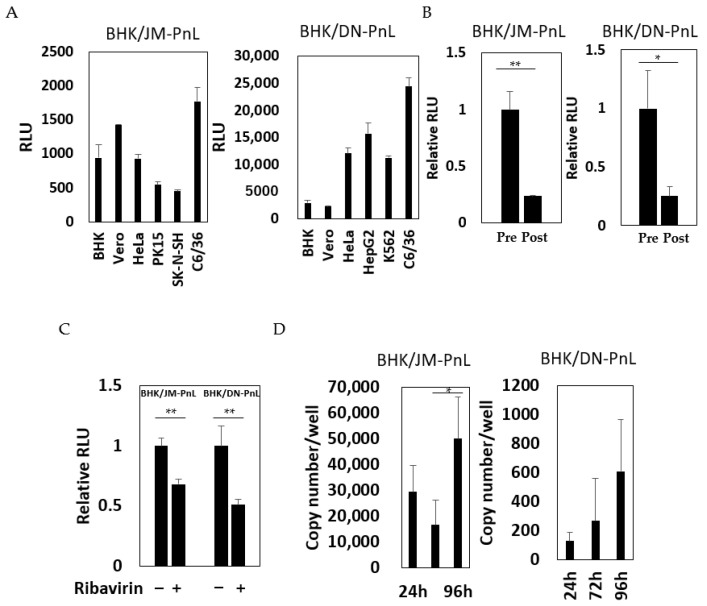
Transfer of subgenomic replicon genomes to various cell lines by EVs. (**A**) Luciferase activities of various cells inoculated with EVs derived from BHK/JM-PnL and BHK/DN-PnL cells were measured at 72 h. Luciferase activities obtained from cells inoculated with naïve BHK cell-derived EVs were used as a negative control and subtracted from each luciferase activity (*n* = 3). (**B**) Culture supernatants before EV isolation (Pre) and EV-depleted culture supernatants (Post) from BHK/JM-PnL and BHK/DNPnL cells were inoculated on BHK cells (*n* = 3). (**C**)Luciferase activities of the BHK cells inoculated with EVs in the presence/absence of ribavirin were measured (*n* = 3). (**D**) The JM-PnL and DN-PnL replicon genomes in the EV-inoculated C6/36 cells were quantified by real-time PCR (*n* = 3). * *p* < 0.05, ** *p* < 0.01.

In order to test whether the replicon RNA associated with the EVs could be transferred to other cells, the EV samples prepared from the culture supernatants of BHK/JM-PnL and BHK/DN-PnL cells were inoculated onto various recipient cells. Luciferase activities were detected in all of the recipient cell lines tested (Figure 4A), suggesting that the replicon RNA could be transferred by EVs. To confirm that the detected luciferase activities resulted from the transfer of EV-associated replicon RNA, the EV-depleted culture supernatants of BHK/JM-PnL and BHK/DN-PnL cells were inoculated onto BHK cells. The luciferase activities of the cells with the EV-depleted culture supernatants were decreased, indicating that EVs could mediate cell-to-cell transfer of the replicon RNA genomes (Figure 4B). To further examine if luciferase activities detected in the EVs-inoculated cells resulted from replication of the transferred replicon genomes, the effects of ribavirin on the luciferase activities in the EV-inoculated cells were examined and replicon genomes in the EV-inoculated cells were quantified by real-time PCR. Ribavirin treatment reduced luciferase activities (Figure 4C) and the replicon genomes in the recipient cells were increased (Figure 4D), showing that transferred replicon genomes were replicable. Those results indicated the possibility that replicon RNA could be transferred from BHK/JM-PnL and BHK/DN-PnL cells to the cells of various origins in an EV-mediated manner.

**Figure 5:**
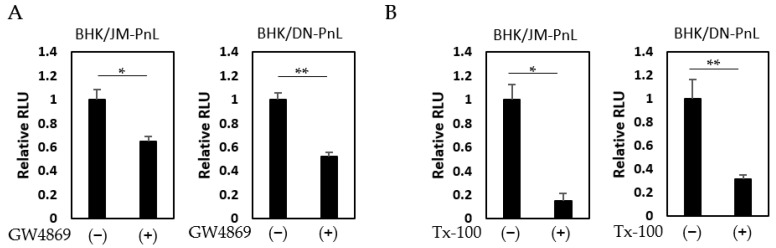
Cell-to-cell transfer of subgenomic replicon RNA was mediated by EVs. (**A**) EVs obtained from BHK/JM-PnL and BHK/DN-PnL cells treated with GW4869 were inoculated to BHK cells (*n* = 3). (**B**) BHK/JM-PnL cell- and BHK/DN-PnL cell-derived EVs treated with Tx-100 were inoculated onto BHK cells (*n* = 3). Data were expressed as relative luminescence units to negative control. * *p* < 0.05, ** *p* < 0.01.

To confirm this possibility, EVs prepared from the culture supernatants of BHK/JM-PnL and BHK/DN-PnL cells that had been treated with the EV production inhibitor, GW4869 (neutral sphingomyelinase) known to inhibit EV production, were inoculated onto BHK cells. The luciferase activities in the recipient BHK cells inoculated with EVs from GW4869-treated donor cells were significantly lower compared with those in BHK cells inoculated with EVs from the untreated donor cells (Figure 5A). Because GW4869 did not affect the replication of the JM-PnL and DN-PnL (Figure S1), the reduction in the luciferase activities (Figure 5A) resulted from the lower production of EVs. Furthermore, treatment of the EV samples with Tx-100, which disrupts the EV membrane, also reduced luciferase activities (Figure 5B). These results clearly demonstrate that EVs are involved in the cell-to-cell transfer of the replicon genome RNA.

### 3.4. Cellular Factors Involved in the EV-Mediated Transfer of the Replicon Genome

**Figure 6:**
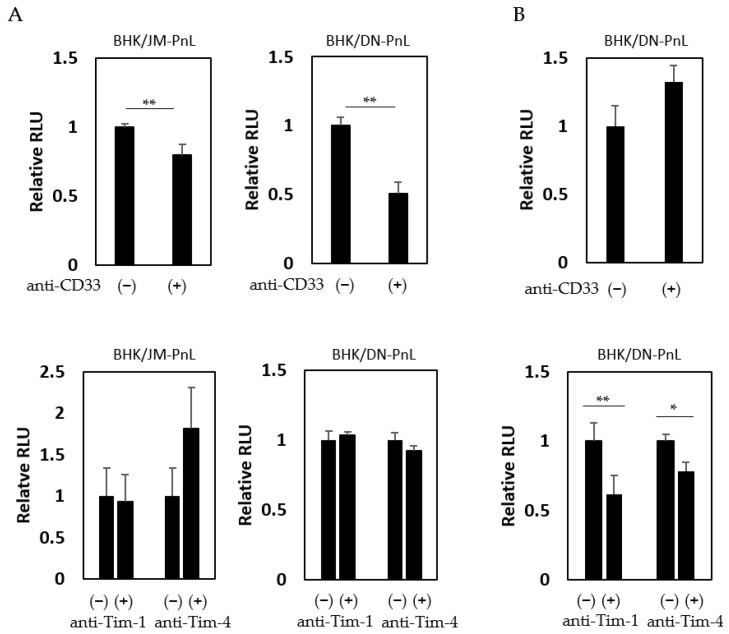
Interaction of EV with cell surface molecules. (**A**) HeLa cells were treated with anti-CD33, anti-Tim-1, and anti-Tim-4 antibodies and then inoculated with EVs derived from BHK/JM-PnL or BHK/DN-PnL cells. (**B**) K562 cells were treated with anti-CD33, anti-Tim-1, and anti-Tim-4 antibodies and then inoculated with EVs derived from BHK/DN-PnL cells. Data were expressed as relative luminescence units to negative control treated with H_2_O (*n* = 3). * *p* < 0.05, ** *p* < 0.01.

It has previously been shown that cell surface proteins, such as CD33, Tim-1, and Tim-4, are involved in EV-mediated transfer of cargo molecules [26]. To examine if these proteins are involved in the EV-mediated transfer of the replicon genome demonstrated in this study, HeLa cells were inoculated with EVs derived from BHK/JM-PnL and BHK/DN-PnL cells in the presence of the anti-CD33, anti-Tim-1, or anti-Tim-4 antibodies. Luciferase activities in the recipient cells were decreased when they were inoculated with the EVs in the presence of the anti-CD33 antibody but not the other antibodies (Figure 6A), suggesting that CD33 might be involved in the EV-mediated transfer of the replicon genome into HeLa cells. On the other hand, when K562 cells were used as the recipient cells, anti-Tim-1 and anti-Tim-4 antibodies but not anti-CD33 antibodies, reduced luciferase activities (Figure 6B), suggesting that Tim-1 and Tim-4 rather than CD33 might be crucial for the EV-mediated transfer of the replicon genome into K562 cells.

**Figure 7:**
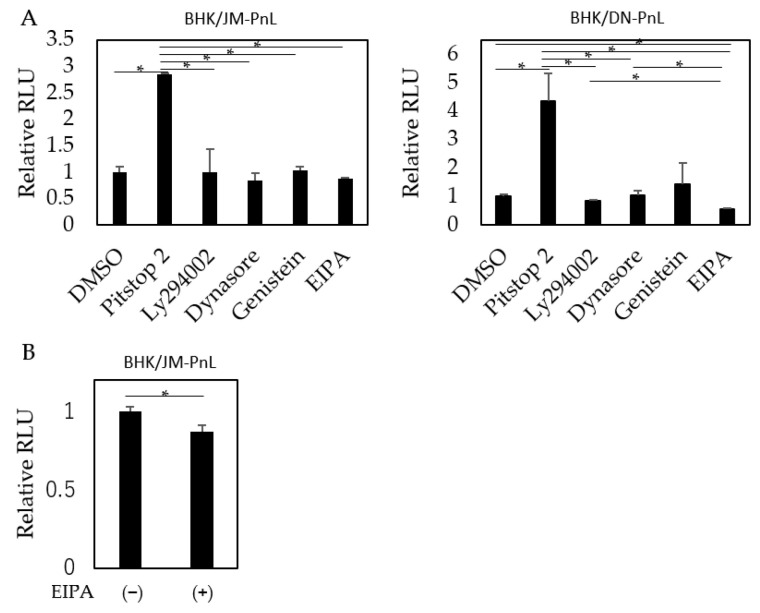
Effects of various entry inhibitors on EV uptake. (**A**) HeLa cells were treated with Pitstop 2, LY294002, dynasore, genistein, or EIPA and then inoculated with EVs derived from BHK/JM-PnL and BHK/DN-PnL cells (*n* = 3). (**B**) HeLa cells were treated with EIPA and then inoculated with EVs derived from BHK/JM-PnL cells for direct comparison (*n* = 5). Data were expressed as relative luminescence units to the negative control. * *p* < 0.05.

To elucidate the pathway by which the replicon genome is internalized into the recipient cells, HeLa cells were treated before EV inoculation with Pitstop 2, dynasore, and genistein, which inhibit clathrin-, dynamin-, and caveolae-mediated endocytosis, respectively; LY294002, which inhibits clathrin-independent endocytosis; or EIPA, which inhibits macropinocytosis. The results showed that luciferase activities in the recipient cells were significantly decreased when the cells were pretreated with EIPA (Figure 7A,B). Interestingly, pretreatment of the recipient cells with Pitstop 2 markedly increased luciferase activities (Figure 7).

## 4. Discussion

EVs, which carry biomolecules, have been shown to play a variety of physiological roles. For example, exosomes have been shown to enhance immune responses by delivering antigen-presenting molecules [27] and DNA [28]. Evidence is also accumulating that EVs are involved in the pathogenesis of various diseases. For instance, exosomes appear to promote tumor growth and metastasis by delivering molecules, such as TGF-β [29], EGFR [30], and miRNAs [31,32]. In addition, recent studies using virus-infected cells have demonstrated the incorporation of viral proteins, as well as viral and cellular RNAs, into EVs, suggesting the possibility that EVs could be involved in viral propagation, pathogenesis, and immune evasion [33,34,35,36,37,38,39,40,41,42]. Several studies reported that EVs could transfer the full-length genome of certain viruses including flaviviruses and that virus dissemination might be mediated by EVs in addition to the classical receptor-mediated infection [13,14,15,16,17,19,20,21]. However, EVs derived from virus-infected cells were used in those previous studies. Therefore, the possibility cannot be excluded that the observed phenomena were due to contamination of the infectious viral particles in the EV samples. In contrast, the cells that harbor flavivirus-derived subgenomic replicons do not produce infectious viral particles; the EVs used in this study were prepared from the culture supernatants of those cells. Therefore, this is the first study that unequivocally demonstrates the ability of EVs to mediate cell-to-cell transfer of flavivirus genome-derived RNA.

Since the ability of EVs to endow the luciferase activity to other cells was resistant to RNase A treatment, the replicon genome was likely packaged inside of the EVs (Figure 3B). The mechanism by which the replicon genome is packaged in the EVs is still unknown. It has been reported that RNA-binding proteins, such as the heterogeneous nuclear ribonucleoprotein family and the Y-box binding protein 1, contribute to the packaging of cellular RNAs into exosomes [43]. Since these cellular proteins have been reported to bind to the genome RNA of JEV and DENV [44,45,46], similar mechanisms might be involved in the incorporation of flavivirus-derived replicon genome into EVs. Figure 2B suggested that the amount of full-length replicon genome of JEV in the EVs seems to be smaller than DENV since the band corresponding to the luciferase-JEV NS1 fragment was faint. Those results implied that the packaging efficiencies of the replicon genome into EVs might be dependent on the virus sequences, which could affect the recruitment of RNA-binding proteins in the host cells to carry the viral gnomes into EVs.

In addition to the replicon genome, proteins reactive with the anti-NS1 protein were detected in the EVs and part of the proteins were shown to be packaged inside of the EVs (Figure 3C,D). The molecular weight of the detected proteins was slightly higher than that of the NS1 protein expressed in the virus-infected cells. Although the actual molecular nature of the detected protein is unknown, it is possible that it corresponds to an incompletely cleaved viral precursor protein, which contains the NS1 epitope. Alternatively, NS1 protein packaged in EVs might be modified because previous studies have demonstrated that proteins found in EVs went through post-translational modification, such as ubiquitination, SUMOylation, or UBL3 modification [47,48,49,50]. The NS2B-NS3 complex was detected in the EVs produced by BHK/DN-PnL cells but not BHK/JM-PnL cells. The reason for the discrepancy is unknown. Flavivirus nonstructural proteins are known to play roles in virus replication and immune evasion [5]. Although the precise abundance of the viral proteins incorporated in the EVs is unknown, it is possible that those viral proteins may contribute to the formation of the EVs containing the replicon genome and to the replication of the replicon genome in the recipient cells.

In this study, treatment of recipient HeLa cells but not K562 cells with the anti-CD33 antibody negatively affected the EV-mediated transfer of the flavivirus-derived replicon genome. This is consistent with the previous study showing that CD33 was involved in the exosome-mediated uptake of fluorescent dye into HeLa cells [51]. In contrast, treatment of recipient K562 cells but not HeLa cells with anti-Tim-1 and Tim-4 antibodies was shown in this study to decrease the exosome uptake. Tim-1 and Tim-4 are members of the T cell immunoglobulin and mucin-domain-containing proteins family and known to bind to phosphatidylserine [52]. While Tim-4 is primarily expressed on antigen-presenting cells, Tim-1 is expressed on various epithelial cells in addition to immune cells [52,53]. It is suggested that receptors for EV uptake are recipient cell-dependent and/or EV producer cell-dependent. Furthermore, it cannot be ruled out that receptors for viral proteins detected in EVs of those on the surface of the EVs play roles in uptake into the recipient cells.

Following the attachment to the surface of recipient cells, EVs are usually internalized into those cells. It has been shown that various mechanisms including endocytosis, macropinocytosis, phagocytosis, and membrane fusion could be involved in the internalization process [54,55,56]. In this study, EIPA treatment of the recipient HeLa cells appeared to negatively affect the internalization of the EVs. Therefore, it is likely that macropinocytosis is involved in the internalization of the EVs carrying the flavivirus-derived replicon into HeLa cells. Apparently, the results contradict the previous studies, which demonstrated clathrin-dependent endocytosis as the major pathway for internalization of the EVs bearing the full-length flaviviral genome [19,21], although partial inhibition of internalization by EIPA treatment observed in this study indicated that other untested internalization pathways might be involved in addition to macropinocytosis. However, EV samples prepared from the virus-infected cells were used in those studies. In addition, neuronal cells were used as the recipient. These differences might be responsible for the apparently incompatible results. Alternatively, the internalization pathway might be dependent on the size of the EVs. Macropinocytosis is known to uptake larger substances (up to 5 µm) than clathrin-dependent endocytosis (~100 nm) [57]. The EVs prepared from flavivirus-infected cells (about 80 to 120 nm on average) [58,59] were larger than those prepared from the replicon-harboring cells in this study (about 40 nm). Therefore, depending on the recipient cell type and the size of the EV, the internalization pathway might be different. 

Interestingly, treatment of the recipient cells with Pitstop 2, which inhibits clathrin-dependent endocytosis, markedly enhanced the internalization of the EVs in our system (Figure 7). Although the exact mechanism for this enhancement is unknown, it is possible that inhibition of clathrin-dependent endocytosis by Pitstop 2 led to the activation of other internalization pathways through the mechanism known as cross-regulation [60]. A similar mechanism was speculated in a previous study where treatment of the recipient cells with chlorpromazine, which inhibits clathrin-dependent endocytosis, increased the EV uptake [61]. 

In conclusion, this study demonstrates that the cells harboring the flavivirus-derived subgenomic replicon, which can avoid contamination of infectious virus particles, serve as a useful EV donor. By using this system, we successfully provided evidence that the RNA molecules derived from JEV and DENV genomes can be transferred from cell to cell in an EV-mediated manner. It was also indicated that the cellular proteins, such as CD33, Tim-1, and Tim-4, are likely involved in the attachment of the EVs on the recipient cell surface. Furthermore, macropinocytosis was shown to play a major role in the EV uptake by the recipient cells in our system. In addition to the classical receptor-mediated infection, EV-mediated cell-to-cell transmission of the flavivirus genome, which could escape the effects of neutralizing antibodies, may play some important roles in viral propagation and pathogenesis. Further studies to elucidate the significance of the EV-mediated transfer of flaviviral components are necessary.

## Data Availability

The data presented in this study are available on request.

## Supplementary Materials

The following supporting information can be downloaded at: https://www.mdpi.com/article/10.3390/v16040524/s1, Figure S1: Effects of GW4869 on the replication of JM-PnL and DN-PnL in BHK cells.
