# Supplementary material for: Dissemination of the Flavivirus Subgenomic Replicon Genome and Viral Proteins by Extracellular Vesicles
Source: Viruses. 2024 Mar 28;16(4):524. doi: 10.3390/v16040524 (PMC11054737; doi:10.3390/v16040524)
Supplement: Supplementary file 1 [file viruses-16-00524-s001.zip › viruses-2923719-supplementary.pdf]

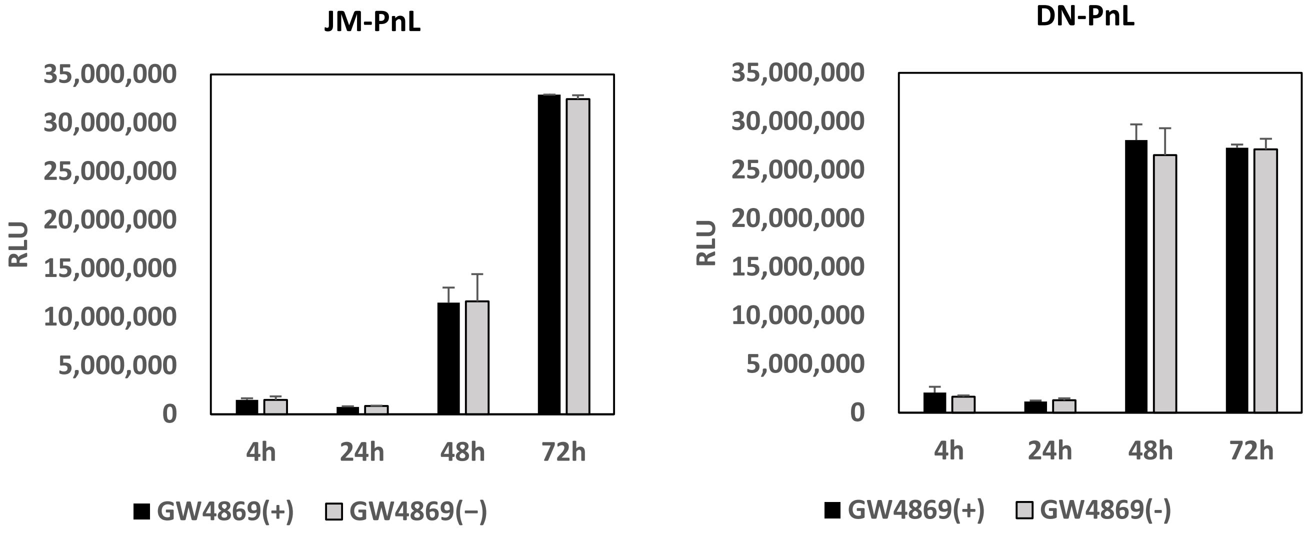

Figure S1. Effects of GW4869 on the replication of JM-PnL and DN-PnL in BHK cells. BHK cells electroporated with JM-PnL or DN-PnL were maintained in the presence/absence of GW4869. Cells were lysed at the indicated time points and those luciferase activities were measured (n=3). Data were expressed as relative luminescence unit to negative control.
